# Design and Fabrication of a Low-Cost Thermopile Infrared Detector

**DOI:** 10.3390/mi12091134

**Published:** 2021-09-21

**Authors:** Ting Liang, Yihao Guan, Cheng Lei, Xuezhan Wu, Yuehang Bai, Jijun Xiong, Lei Qi

**Affiliations:** 1 School of Instrument and Electronics, North University of China, Taiyuan 030051, China; liangtingnuc@163.com (T.L.); guanyih19@163.com (Y.G.); wxz110@126.com (X.W.); baiyuehang1109@163.com (Y.B.); xiongjijun@nuc.edu.cn (J.X.); 2 North Automatic Control Technology Institute, Taiyuan 030006, China; 18635113272@163.com

**Keywords:** MEMS, infrared detector, thermocouple, closure film, passivated absorption layer

## Abstract

In this paper, we design and optimize a low-cost, closed-film structure of a microelectromechanical systems (MEMS) thermopile infrared detector. By optimizing the circular arrangement of thermocouple strips and the thermal isolation design of the cold end to pursue a higher temperature difference, in addition to eliminating the absorption region, silicon nitride is deposited on the whole device surface as a passivated absorption layer. This reduces the cost while maintaining the voltage response and is suitable for mass production. The optimized detector had a 22.6% improvement in the response rate to 34.2 V/W, a detection rate of 1.02 × 10^8^ cm·Hz^1/2^/W, and a response time of 26.9 ms. The design optimization of this detector provides a reference for further development of IR detectors.

## 1. Introduction

All objects above absolute zero emit infrared radiation, and by measuring infrared radiation the temperature of the measured object can be accurately obtained under non-contact conditions. Depending on the method of transformation of the infrared radiation signal, detectors can be divided into photonic and thermal detectors [1]. Among thermal detectors, thermopile infrared detectors have been widely studied and used because they can operate at room temperature, do not require chopping and bias voltage, have a wide spectral response range, and are simple and convenient to operate [2]. Thermopile infrared detectors can transform the infrared radiation emitted by external objects into electrical signals through the Seebeck effect, such that infrared thermopiles can monitor temperature changes through convection and radiation. The most common is carried in the ear temperature gun used to measure human body temperature; it can detect the radiation energy transmitted from liquids and gases in the form of infrared radiation. In addition to this it can be used widely in smart homes, automotive electronics, industrial automation and other noncontact temperature measurement scenarios, where one can accurately monitor the temperature of various heat sources from a distance. It can even achieve the purpose of imaging through arrays.

Thermopile chips generally consist of a silicon window substrate, a suspension support layer, a thermocouple strip structure, and an absorption zone [3]. The thermopile is based on the Seebeck effect, which means that two different semiconductor materials are connected in series, and the two ends are in different temperature environments, and because the temperature difference cause the carriers in the semiconductor material to migrate, this generates a voltage [4]. The cold end of the thermocouple strip is located on the silicon window substrate, and the hot end is usually covered with a separate absorber layer, which is thermally isolated by etching to make the support film layer isolated from the silicon substrate. Therefore, one way to pursue high output performance of thermopiles is to increase the temperature difference. Covering the hot end with different types of absorber materials is a common method to improve the performance of thermopiles. Shakeel Ashraf integrated the SU-8 into the absorber structure of the thermopile detector, which leads to an improved absorption rate and improved performance [5]. Ting-Wei Shen improved thermopile response by a factor of 2.6 by embedding an umbrella-shaped absorber [6]. However, such individual absorption structures are usually more complicated to prepare, poorly compatible with the preparation processes, have a high cost, and are not suitable for mass production. Silicon nitride is generally used as an absorbing material because of its infrared absorption effect and narrow absorption range [7].

In this study, we optimize the design of the thermopile chip by improving the temperature difference by adjusting the area of the absorption zone on the suspension film, the spacing between the hot and cold ends of the thermocouple strip, and the design of the ohmic contact zone at the cold end. Silicon nitride is used as the passivation absorber layer and additional IR absorbing material is eliminated to reduce the cost. Finally, we measure the thermopile sensor before and after optimization.

## 2. Principle

In this paper, we have chosen a circular suspended membrane structure for our structure, and the specific structural design is described in detail below.

**Figure 1:**
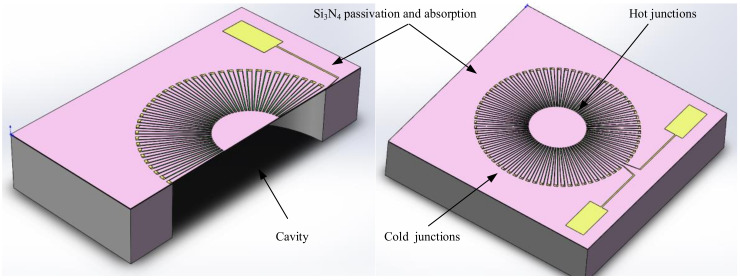
Schematic diagram of the designed and prepared circular thermopile detector.

As shown in Figure 1, when external infrared radiation is irradiated onto the thermopile chip, a temperature difference ($T_{diff}$) is generated between the hot end in the center of the suspended film, which absorbs heat, and the cold end on the edge silicon substrate. According to the Seebeck effect, a temperature difference electric potential (U) is generated between the hot and cold ends and its mathematical expression is [8]:

(1) $$U=NT_{diff}\alpha_{a-b}$$

where N is the number of thermocouple strip pairs, $T_{diff}$ is the temperature difference from the hot end to the cold end in the thermopile, and $\alpha_{a-b}$ is the difference between the Seebeck coefficients of two different materials. According to Equation (1), the response voltage can be increased by increasing the number of thermocouple strip pairs and increasing the temperature difference.

In addition, some important parameters of the thermopile need to be characterized, mainly the detector responsivity $R_{v}$, detection rate $D^{*}$, response time τ, and thermopile resistance R.

The responsivity is generally characterized by the ratio of the response voltage to the IR radiated power, and usually that is expressed as [9,10]:

(2) $$R_{v}=\frac{U}{P_{0}}=\frac{U}{\phi_{0}A_{d}}$$

where *P*_0_ is the infrared radiated power, *A_d_* is the absorption area of the device, *φ*_0_ is the infrared radiated power density, and *φ*_0_ is generally denoted as [11]:

(3) $$\phi_{0}=\frac{C_{r}\sigma \varepsilon_{1}\left(T_{1}^{4}-T_{0}^{4}\right)A_{S}}{\pi d_{0}^{2}}$$

where *C_r_* is the root mean square conversion factor, *σ* is the Stephen Boltzmann constant, *ε*_1_ is the emissivity of the blackbody, *A_s_* refers to the area of the blackbody, *d*_0_ is the distance from the blackbody to the detector surface, *T_1_* is the blackbody temperature, and *T*_0_ is the ambient temperature.

The specific detection rate can express the detection capability of the thermopile device, which can usually be calculated by the equation [2]:

(4) $$D^{*}=\frac{R_{v}\sqrt{A_{d}\Delta f}}{U_{n}}$$

where Δf is the test bandwidth, generally taken as 1 Hz, and *U_n_* is the noise voltage of the IR sensor which can be calculated by the formula [4]:

(5) $$U_{n}=\sqrt{4kR_{0}T_{0}\Delta f}$$

In addition to this, the total resistance value of the thermopile is also a design indicator, and the resistance in the thermopile consists mainly of the sum of the resistance values of all thermocouple strips of two different materials, which can be generally expressed as [12]:

(6) $$R=N\sum_{i=1}^{2}\gamma_{i}\frac{l_{i}}{d_{i}w_{i}}$$

where, $\gamma_{i}$ is the resistivity of the thermocouple strip, $l_{i}$, $d_{i}$, and $w_{i}$ represent the length, thickness and width of the thermocouple strip material, respectively.

## 3. Structural Design and Optimization

Since circular membranes are less stressful than square membranes, the structure of the thermopile is designed and optimized based on a circular arrangement.

### 3.1. Absorption Area Design

**Figure 2:**
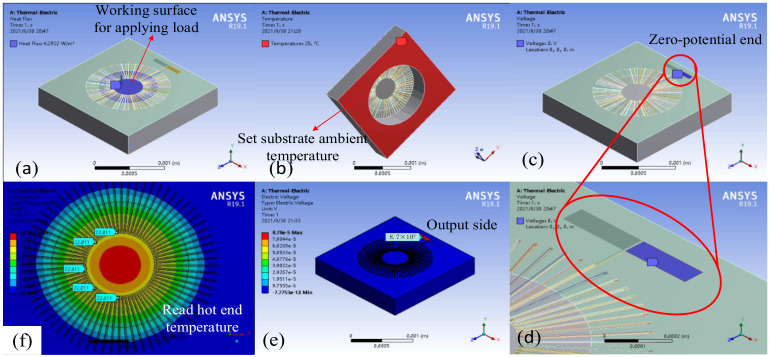
Finite element simulation setup. (**a**) Changing the absorption area by changing the applied load working surface. (**b**) Adding room temperature to the silicon substrate ground. (**c**) Adding zero potential at one end of the aluminum strip. (**d**) Enlarged zero-potential addition surface. (**e**) The location of the high potential after simulation calculations, read by the probe. (**f**) Temperature distribution cloud, calculated by reading the temperature of all hot ends and cold ends through the probe to obtain the temperature difference.

As shown in Equation (1) in Section 2, the response voltage of the thermopile is related to the difference in the Seebeck coefficient of the material. The material of the circular thermopile is therefore composed of P-polySi and Al, which guarantees a reliable Seebeck coefficient difference and a certain degree of cost saving. We distribute the thermocouple strip uniformly in a circle at a certain angle, and first simulate it by changing the area of the absorption zone. Through ANSYS thermoelectric coupling simulation, as shown in Figure 2, all structures and materials are kept constant, and a fixed heat flux load is applied to the silicon nitride above the cavity. By varying the working area of the silicon nitride, we simulated the effect of different absorption areas on the temperature and output potential. With a temperature of 25 °C applied to the bottom surface of the silicon liner and a low potential of 0 V set for the pads connected to the aluminum strip, we read the simulated temperature at all thermal junctions and the simulated maximum potential at the polysilicon connections with a probe.

**Figure 3:**
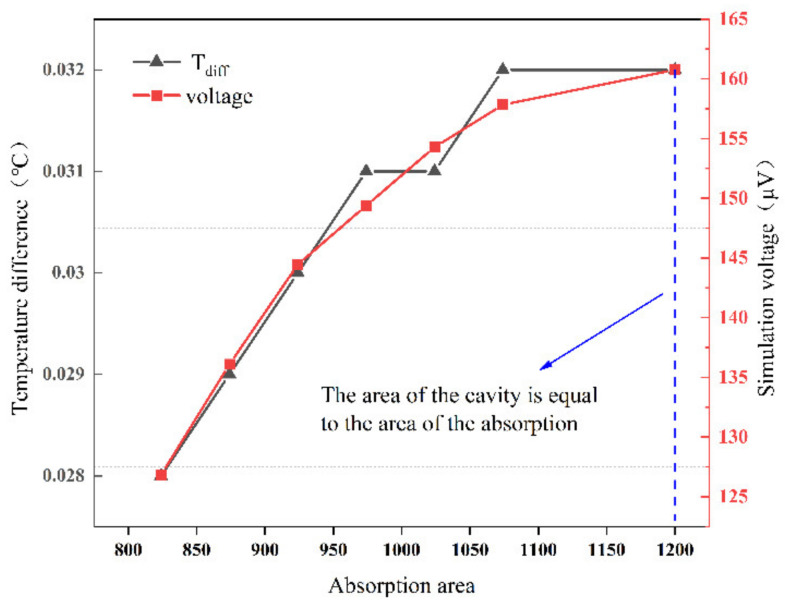
Effect on simulated output voltage with area of the absorption zone.

As shown in Figure 3, according to the simulation results, it can be seen that the response is improved as the area of the absorption zone increases, and the simulation output is maximized when the area of the absorption zone is equal to the area of the back cavity. Therefore, we considered depositing a layer of silicon nitride material on the entire surface of the thermopile. This silicon nitride acts both as an absorber and as a passivation protector. In contrast, conventional thermopiles generally consist of a silicon substrate, a support layer, a passivation layer, a thermopile and an absorber [3]. This reduces the process difficulty to some extent.

The actual device is the entire surface that will absorb infrared radiation, where the heat absorbed above the silicon substrate is transferred directly down to the substrate causing losses, resulting in temperature differences only in the infrared radiation absorbed by the silicon nitride above the cavity. Therefore, we applied the heat flux to the entire surface in the subsequent optimization of the thermocouple strip arrangement when simulating the applied load, while the area of the absorber was considered equal to the area of the cavity in the theoretical calculation. To facilitate theoretical calculations, we generally ignored the Gaussian profile caused by external radiation, and by default the radiation irradiated to the surface of the absorber is uniform.

### 3.2. Thermopile Structure Design

First, a thermocouple strip with 72 pairs and an angle of 5° between each pair of thermocouple strips was designed as a reference thermopile according to the resistance requirements (Reference thermopile named YUAN-72). According to Equation (1), one of the ways to increase the output voltage is to increase the temperature difference. Then we performed thermal simulations for the circular thermopile structure, with temperature scattering transfer in all directions along the thermocouple strip. Therefore, we considered extending the hot end towards the center, so that the hot end would be in a higher temperature range, in addition to adding a certain number of thermocouple strips.

According to Seebeck’s principle, the carriers in the thermocouple strip flow from high to low temperatures. The main principle of our optimization is to ensure that the flow direction of the carriers in the thermocouple strip is, as much as possible, in line with the temperature gradient, so that the heat capacity and thermal conductivity of the thermopile device can be reduced to a certain extent and thus a smaller response time can be guaranteed. If the polysilicon and aluminum strips are kept in parallel rows, the parallel pair of thermocouple strips are arranged along a circular scattering-like temperature gradient. Although this ensures that all carriers flow in the same direction as the temperature gradient, the larger hot end and the limited inner circular space will limit the elongation of the hot end toward the higher central temperature.

Therefore, we only arrange the polysilicon strips along the temperature gradient in a scattering pattern, and elongate the hot end as much as possible to the center at a higher temperature, and then connect the hot and cold ends of adjacent polysilicon in series through aluminum. Although the direction of motion of the aluminum strip and the carriers in it did not exactly coincide with the temperature gradient, with the elongation of the length and the increase of the number of thermocouple strip pairs, the angle between the carrier flow direction in the aluminum and the temperature gradient is further reduced, thus ensuring that the temperature gradient and the carrier flow direction are as consistent as possible.

**Figure 4:**
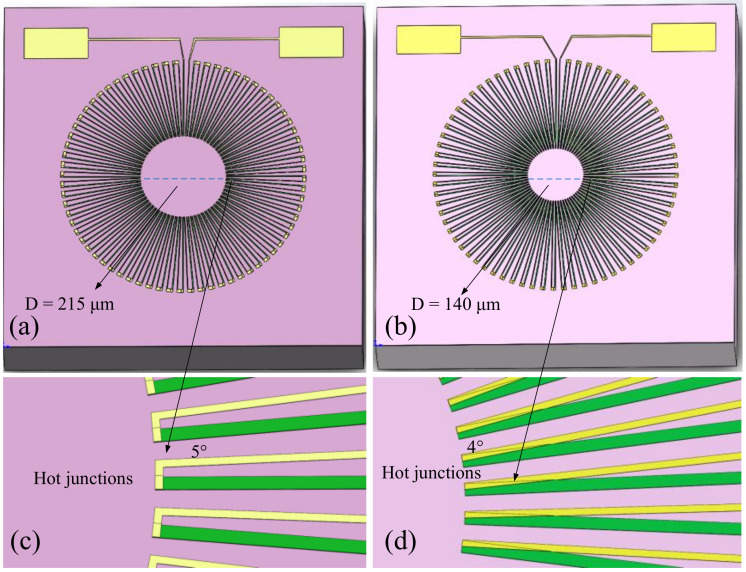
Infrared detector of the thermopile before and after optimization. (**a**) Reference thermopile before optimization. (**b**) Optimized thermopile. (**c**) Hot end design of the reference thermopile, (**d**) Hot end design of the optimized thermopile.

As shown in Figure 4, the number of pairs of thermocouple strips in the optimized circular thermopile is 90 pairs, and the angle between thermocouple strips is reduced to 4°. The distance between hot ends decreases sharply after elongation, so the ohmic contacts at the hot ends are cross-connected. The diameter of the circle formed by all hot ends is reduced from approximately 215 to 140 μm (The optimized thermopile is named YUAN-90).

**Figure 5:**
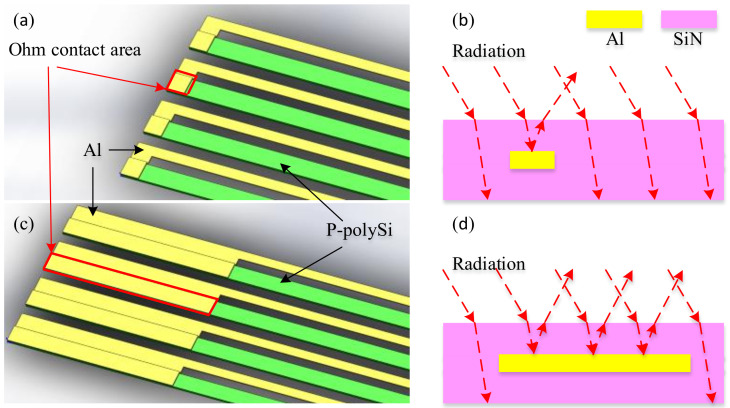
Cold end design. (**a**) Conventional cold end with small ohmic contact area. (**b**) Conventional cold end thermal isolation mechanism, most of the radiation is absorbed by the surface silicon nitride, there is almost no reflection. (**c**) Optimized cold end design with the same effective length of thermocouple strip, increased ohmic contact area, and increased area of aluminum. (**d**) The optimized cold end thermal isolation mechanism, compared with preoptimization, part of the radiation is first absorbed by the surface silicon nitride and is reflected when it reaches the aluminum layer, thus reducing the cold end absorption to some extent.

In addition, we increased the cold end ohmic contact area of the optimized round thermopile, and the metallic aluminum on top of the cold end ohmic contact area can play a certain degree of reflection effect to improve the performance. As shown in Figure 5, the conventional cold end only needs to connect the thermocouple strips, and there is almost no thermal isolation effect. We leave the effective length of the thermocouple strips unchanged and increase the ohmic contact of the cold end, so the area of aluminum per unit area is enhanced, and the aluminum can reflect the radiation absorbed by the silicon nitride on the surface of the cold end to a certain extent, thus improving the thermal isolation effect of the cold end. This design ensures a lower cost and process difficulty compared to preparing a separate layer of aluminum to cover the cold end surface.

**Figure 6:**
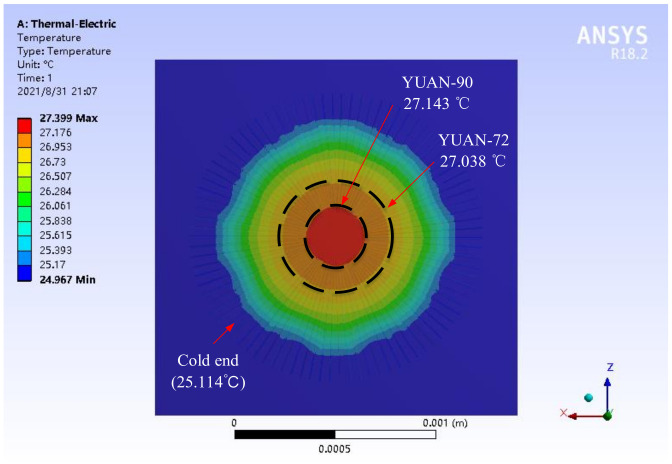
Simulation results of the detector temperature distribution.

The detector was modeled and simulated with Ansys Workbench 19.1. The thermoelectric stack chip is square, the cavity is circular, the load is applied to the upper surface of the support film above the cavity, the radiated power density (*φ*_0_) is 491.472 W/m^2^, the cold junction temperature is 25 °C, and the output electrode potential of the aluminum strip is set to 0 V. As can be seen from the observation of the temperature distribution cloud, the closer to the center, the higher the temperature, and all the simulated temperatures at the hot end are the same. The average temperature difference between the cold junction and the hot junction of the reference thermopile is about 1.924 °C, and the average temperature difference between the optimized cold junction and the hot junction is about 0.014 °C, as shown in Figure 6. The performance of the thermopile obtained after ANSYS simulation is shown in Table 1.

## 4. Preparation of the Detector

In this work, we designed a circular thermopile infrared detector without additional absorber region material and processed by a complementary metal oxide semiconductor compatible process, which overall pursues a simple preparation process and low cost.

**Figure 7:**
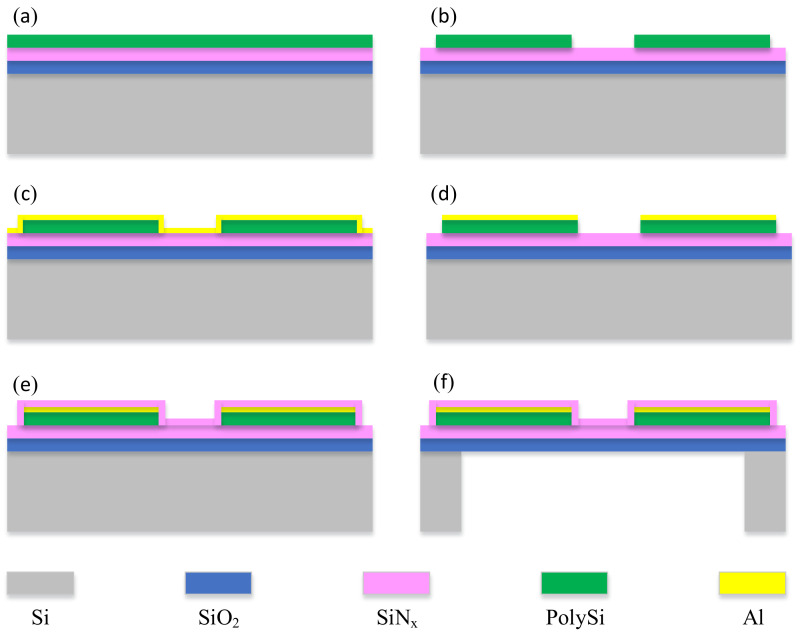
Process flow for the preparation of infrared thermopiles without an additional absorber layer for this circular design. (**a**) Preparation of composite support film structure, (**b**) patterning of polysilicon thermocouple strip, (**c**) sputtering of metallic aluminum, (**d**) formation of aluminum strip by lift-off technology, (**e**) deposition and patterning of silicon nitride passivated absorber layer, (**f**) release of suspended film by dry etching of SF_6_ in the back cavity.

The micromachining process of the thermopile infrared detector is shown in Figure 7. Firstly, the support film layer was prepared by thermal oxidation of 1600 nm silicon oxide and low-pressure chemical vapor deposition (LPCVD) of silicon nitride as the composite support film layer; then 8000 Å polysilicon was prepared by low-pressure vapor deposition and injected with boron ions to obtain the P-polySi layer (Figure 7a). The silicon nitride in the support layer acts as an etch blocking layer, and then the polysilicon thermocouple strip is patterned by photolithographic etching (Figure 7b). The aluminum strips were prepared by lithography, sputtering, and lift-off processes (Figure 7c). The polysilicon and aluminum strips were connected in series at both endpoints, laid flat on the same layer, and annealed at high temperatures to allow the polysilicon to form an ohmic contact with the aluminum (Figure 7d).

Then, plasma-enhanced chemical vapor deposition (PECVD) deposited a silicon nitride film on the upper surface of the thermocouple strip as a passivation protection film for the thermopile, again as an infrared absorbing layer. This film layer is patterned to expose only the metallic aluminum Pad (Figure 7e), and by now all processes on the front side are complete. In order to facilitate batch production, we use the SF6 gas deep etching method to release the membrane. However, this method of releasing the film requires the application of pump oil or paraffin wax on the front side to protect the structure. While the entire thickness of the suspension membrane of this design is only about 2 μm, and with the conventional frontal protection method it is very easy to cause diaphragm breakage. Therefore, in this study, by applying uniform photoresist around the patch, the structural silicon wafer is placed face down on the patch coated with the patch adhesive ring, so that all thermopile structures are not in direct contact with the patch. This protects both the frontal structure during etching and does not cause damage due to external forces when removing the patch to release the film layer, which greatly reduces the film breakage rate (Figure 7f).

**Figure 8:**
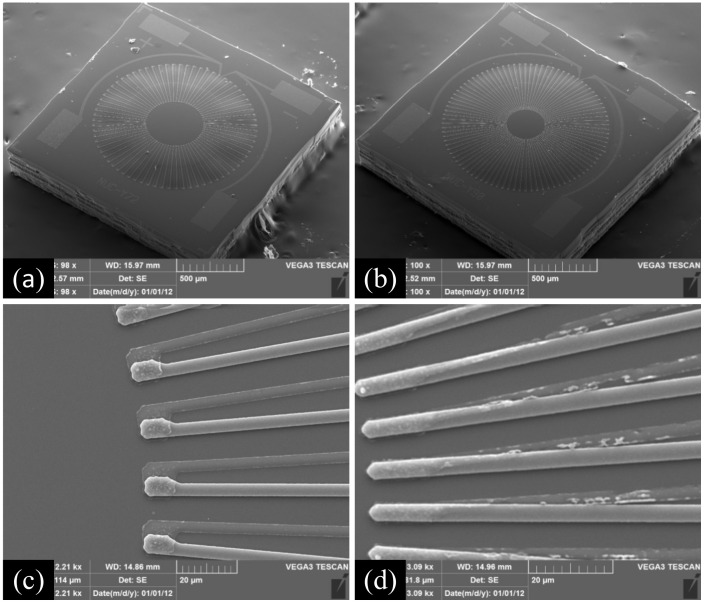
Prepared infrared thermopile detector. (**a**) Reference thermopile detector, (**b**) optimized thermopile detector, (**c**) hot end arrangement of the reference thermopile, (**d**) optimized hot end arrangement of the thermopile.

The thermopile infrared detector designed in this paper was successfully prepared as shown in Figure 8. The size of the prepared detector was 1.8 × 1.8 mm^2^. Since its effective area is only 1.2 × 1.2 mm^2^, the size of our detector can be directly reduced to 1.5 × 1.5 mm^2^ without any change in performance.

**Figure 9:**
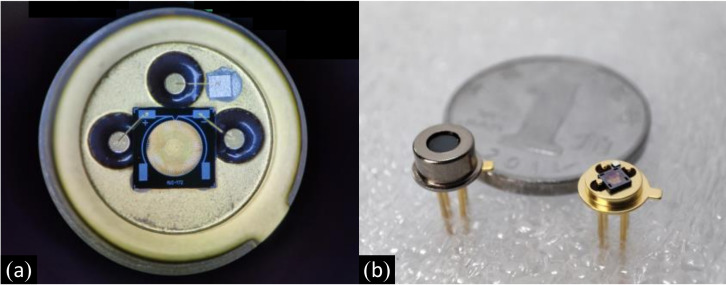
Infrared thermopile detector with TO package. (**a**) Prepared infrared thermopile detector observed under an optical microscope, (**b**) Packaged infrared thermopile detector.

We packaged the prepared thermopile IR detector in a nitrogen environment with TO-46 for subsequent performance testing and analytical verification, as shown in Figure 9.

## 5. Characterization of the Detector

**Figure 10:**
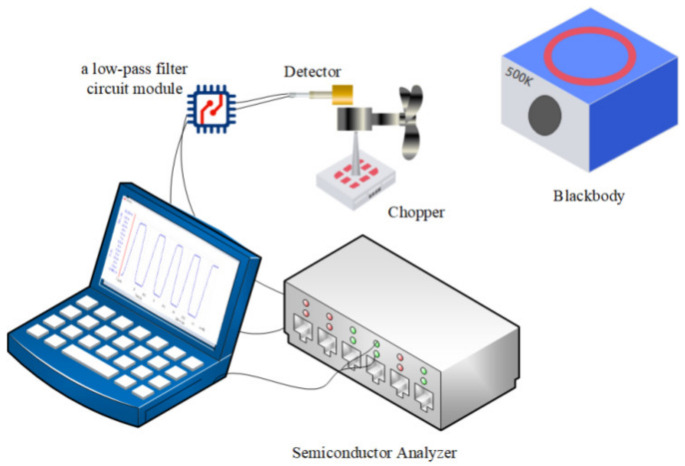
The built infrared thermopile test system.

In order to test the packaged IR thermopile detector, as shown in Figure 10, an IR test system was built. The test system consisted of a blackbody infrared radiation source (HT-C128, Shanghai, China), a chopper controller (SR540), a low-pass filter circuit module, and a B1500A semiconductor analyzer. The TO vacuum package thermopile infrared detector was placed at a distance of 12 cm from the blackbody radiation source, the chopper was placed between the blackbody and the detector, the back end was connected to a low-pass filter circuit to suppress high frequency noise, and finally the semiconductor analyzer was connected to get the test data.

**Figure 11:**
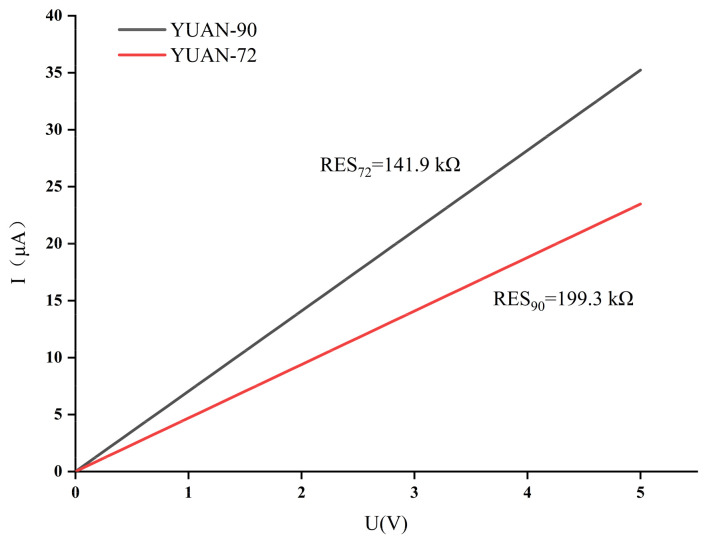
IV characteristic curve test results of infrared detector.

In the test process, the blackbody radiation source temperature was kept at 500 K, and the detector substrate temperature was kept at the same 25 °C as room temperature. The distance between the detector and the blackbody was kept at 12 cm, the chopper was located between the blackbody and the detector, and the chopper frequency was adjusted so that the test waveform appeared as a square wave. The test results of the IV characteristics of the thermopile infrared detector are shown in Figure 11 (YUAN-72 is the reference thermopile, YUAN-90 is the optimized thermopile).

**Figure 12:**
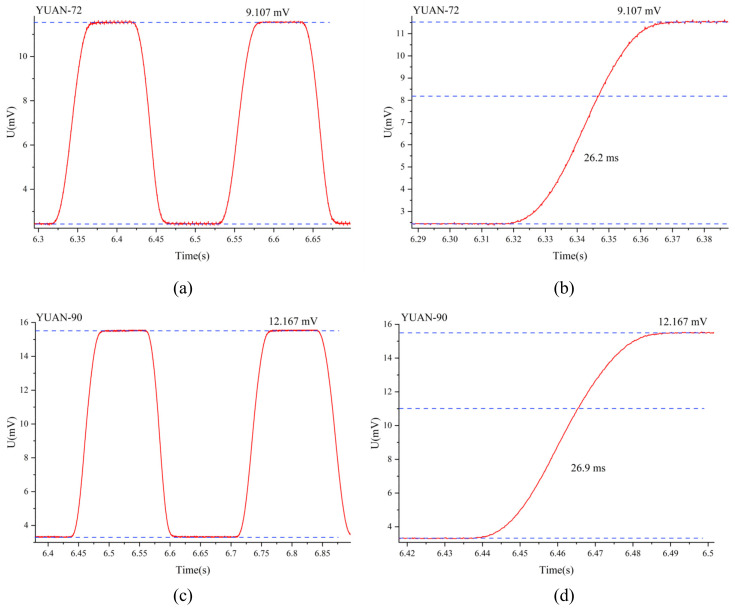
Response of the detector under a black body radiation of 500 K and chopper frequency of 4 Hz. (**a**) Reference thermopile response voltage. (**b**) Rising edge of the reference thermopile amplification. (**c**) Optimized thermopile response voltage. (**d**) Rising edge of the optimized thermopile amplification.

Figure 12 shows the output waveform of the prepared detector for two cycles. The test results show that the response voltage of the optimized detector is 12.167 mV under the condition of 500 K blackbody radiation and at a certain Hertz chopping frequency, and the distance between the detector and the blackbody is about 120 mm. Figure 12d shows the rising edge of the waveform amplification in Figure 12c, which shows its response voltage is 26.9 ms. However, the response voltage measured by adding a chopper has a certain cutoff, and in order to be consistent with the characterization method of commercially available products, we also reduced the distance between the thermopile detector and the blackbody to 30 mm without adding a chopper. We measured the response voltage, took the average of five measurements for each type of device, and characterized the response rate and detection rate. As shown in Table 2, the responsiveness of the optimized device was 34.221 V/W, the detectivity was calculated as 1.02 × 10^8^ cm·Hz^1/2^/W, and the noise voltage was calculated as 37.943 nV/Hz^1/2^. The response voltage obtained after testing was very close to the simulated value obtained from the simulation above, which again verifies the feasibility of the simulation design, and the error in the resistance value was because the hot end of the thermopile is cross-connected by the optimization, which affects the calculation.

**Figure 13:**
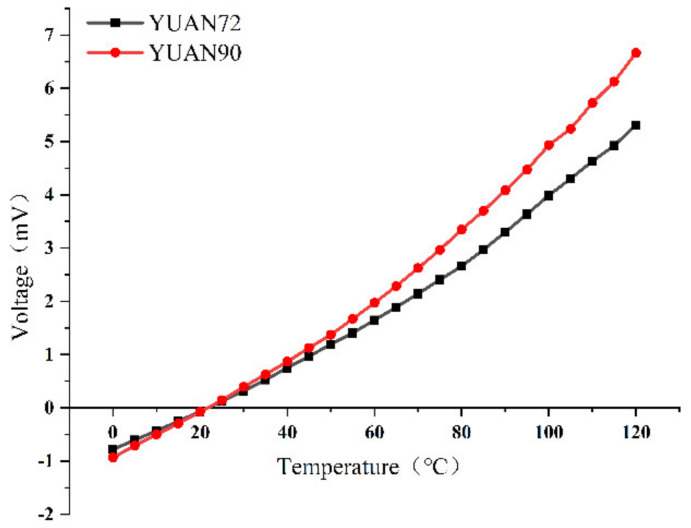
V−T characteristic curve test results of infrared detector.

In addition to this, we also performed temperature response tests for the detector. We fixed the detector at 30 mm in front of the blackbody radiation source (DCN1000L), and the V-T response of the prepared thermopile detector is shown in Figure 13 by varying the blackbody radiation temperature in steps of 5 °C. The results show that the temperature response of the optimized thermopile was improved.

**Figure 14:**
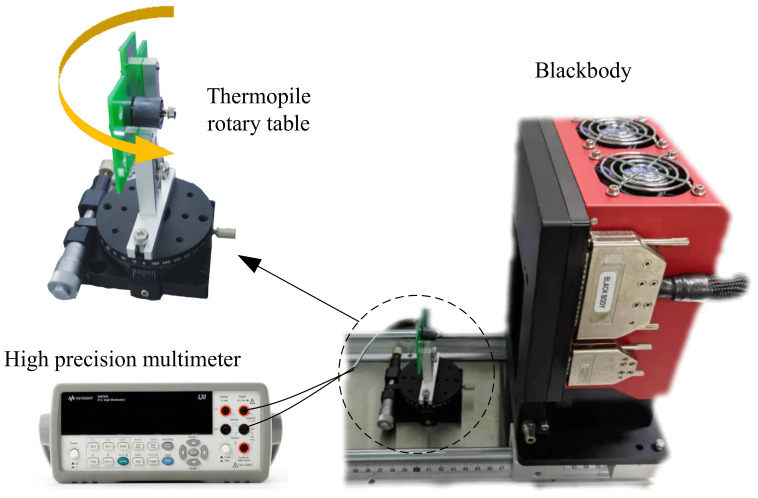
Field of view test system.

**Figure 15:**
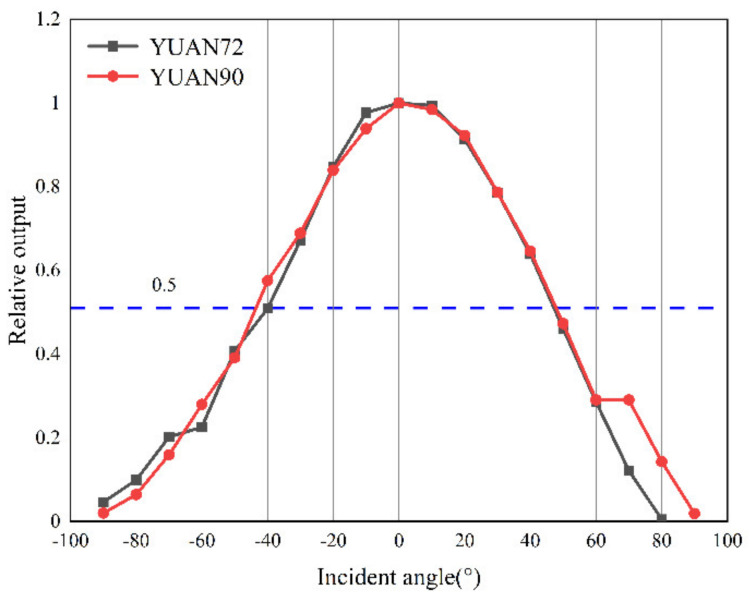
Field of view test results.

Finally, we also characterized the field of view of the packaged thermopile IR detector. A field-of-view test system was built as shown in Figure 14. The measured response voltage was further converted by rotating the thermopile detector to change the relative angle to the blackbody, and the field-of-view test results are shown in Figure 15. The field-of-view of the reference thermopile was 87° at 50% of the peak response rate, and the optimized thermopile detector field-of-view also reached 92°, which was also enhanced.

## 6. Conclusions

In this paper, a low-cost, high-performance circularly arranged infrared thermopile detector is designed, optimized and characterized. To ensure a good cost as well as to obtain a better performance, silicon nitride is deposited on the whole surface as passivation protection and for an IR absorption effect without an additional IR absorption region and absorption material. In addition to this, a thermocouple strip arrangement design optimization idea is provided, i.e., to ensure that the carrier motion in the thermocouple is as consistent as possible with the temperature gradient, and the hot end is as elongated as possible toward the center. At the same time, the cold end design is optimized by increasing the ohmic contact to achieve thermal isolation of the cold end and further ensure a higher temperature difference. The response voltage of the optimized device was largely improved by setting up an infrared test system for characterization. The device’s optimized responsivity was improved by 22.6% to 34.2 V/W under the test conditions in the paper, with a detection rate of 1.02 × 10^8^ cm·Hz^1/2^/W and a response time of 26.9 ms. In this study, the design of thermopile detector with circular structure is optimized to pursue a high voltage response. This optimized thermocouple strip arrangement, absorption zone and cold end design allows for low cost batch production with high response and can be a reference for the further development of thermopile infrared detectors.

## Figures and Tables

**Table 1:** Finite element simulation values of two thermopiles before and after optimization.

Parameter	Setting Parameters	Unit	Reference Thermopile	Optimized Thermopile
Electrical resistance	25 °C	kΩ	143.5	172.1
Temperature difference	/	°C	1.924	2.029
Response Voltage	D: 30 mm	mV	15.09	19.93

**Table 2:** Performance parameters of the prepared thermopile detectors.

Parameter	Test Conditions	Unit	Reference Thermopile	Optimized Thermopile
Electrical resistance	25 °C	kΩ	141.9	199.3
Response Voltage	D: 30 mm	mV	15.518	19.012
Responsivity	D: 30 mm	V/W	27.9	34.2
Specific detectivity	500 K, 8–14 μm, 4 Hz	10^7^ cmHz^1/2^ W^−1^	12.1	10.2
Noise Equivalent Power	/	nW/Hz^1/2^	0.933	1.109
Noise voltage	/	nV/Hz^1/2^	26.057	37.043
Temperature coefficient	/	%/K	0.031	0.038
Field of view	@50% target signal	°	87	92
Response Voltage	D: 120 mm	mV	12.111	15.859
Response time	D: 120 mm, 4 Hz	ms	26.2	26.9
